# Guest Editorial: Papers from the 12th Workshop on Augmented Environments for Computer-Assisted Interventions

**DOI:** 10.1049/htl.2018.5092

**Published:** 2018-10-29

Welcome to this Special Issue of the IET's *Healthcare Technology Letters* (HTL) journal dedicated to the 12th edition of the Joint International Workshop on Augmented Environments for Computer-Assisted Interventions (AE-CAI) and Cerebral Visualization (CereVis). We are pleased to present the proceedings of this exciting scientific gathering held in conjunction with the Medical Image Computing and Computer-Assisted Interventions (MICCAI) conference on September 16th, 2018 in Granada, Spain.

The AE-CAI workshop series dates as far back as MICCAI 2006 in Denmark and has evolved as a forum to discuss the latest developments in Computer-Assisted Interventions (CAI). CAI is a field of research and practice, where medical interventions are supported by computer-based tools and methodologies. CAI systems enable more precise, safer, and less invasive interventional treatments by providing enhanced planning, real-time visualization, instrument guidance and navigation, as well as situation awareness and cognition. These research domains have been motivated by the development of medical imaging and its evolution from being primarily a diagnostic modality towards its use as a therapeutic and interventional aid, driven by the need to streamline the diagnostic and therapeutic processes via minimally invasive visualization and therapy.

To promote this field of research, the AE-CAI workshop series seeks to showcase papers that disseminate novel theoretical algorithms, technical implementations, and development and validation of integrated hardware and software systems in the context of their dedicated clinical applications. This year's workshop is a joint effort between two communities – the AE-CAI community and the CereVis community. We joined forces back in June 2018 in light of our common interests in neuro-navigation and visualization for image-guided neuro-interventions.

The objective of the AE-CAI & CereVis workshop has been to attract scientific contributions that offer solutions to the technical problems in the area of augmented and virtual environments for computer-assisted interventions, with CereVis contributions focusing on neuro-navigation and neuro-interventions, and to provide a venue for dissemination of papers describing both complete systems and clinical applications. The workshop attracts researchers in computer science, biomedical engineering, computer vision, robotics, and medical imaging.

The workshop featured a single track that consisted of long and short oral presentations showcasing original research engaged in the development of virtual and augmented environments for medical image visualization and image-guided interventions. To foster networking and discussion, we also provided all authors with the opportunity to present their work as a poster in addition to their oral presentation slot.

In addition to the contributed papers, we were pleased to welcome two keynote speakers: Dr. Klaus Engel and Dr. Terry Peters. Dr. Engel is an expert in visualization from Siemens Healthineers in Erlangen, Germany and spoke on cinematic rendering from medical imaging. Dr. Terry Peters is a pioneer in image-guided interventions from Robarts Research Institute at Western University in London, Ontario, Canada and spoke on current and future trends in mixed, augmented and virtual reality visualization in medicine.

All manuscripts submitted to the joint AE-CAI & CereVis 2018 were held up to journal standards, as the ultimate objective was to publish accepted work in this Special Issue of the IET's *Healthcare Technology Letters* journal. This year AE-CAI & CereVis received 20 manuscripts spanning a strong geographic representation from Europe, North America and Asia. The review process was rigorous and involved evaluation of each manuscript by three to five external reviewers. Following the first stage of review, sixteen manuscripts were further considered for major or minor revision, and resubmission. Authors were required to submit a response to reviewers and resubmit a revised manuscript that addressed all reviewers’ critiques.

Once revised, all resubmitted manuscripts entered a second round of review conducted by the AE-CAI Program Committee, Workshop Chairs, as well as the HTL Managing Editor and Editor-in-Chief to ensure that all reviewers’ critiques were properly addressed in the revised manuscripts and that the quality of the manuscript was appropriate for journal publication. All manuscript were then accepted for publication and forwarded to the journal for production and publication.

On behalf of the AE-CAI & CereVis 2018 Program and Organizing Committee, we would like to extend our sincere gratitude to all authors, presenters, and attendees for their scientific contribution, enthusiasm, and support. We also extend special thanks to all reviewers for providing detailed and timely critiques of the submitted manuscripts. We also acknowledge the support we received from the MICCAI 2018 Workshop Committee, and the HTL Editorial Office for their dedication to maintaining the high quality of AE-CAI and CereVis workshops, and for selecting outstanding manuscripts that fostered exciting discussions at the meeting.

Last but not least, we acknowledge our generous sponsors. We thank *Northern Digital Inc. (NDI)* for their continued support over the years that has enabled us to recognize AE-CAI authors for their much deserved dedication and scientific enthusiasm through several paper awards. We also thank *BrainLab* for their generous sponsorship of the top papers presented as part of the neuro-imaging/navigation/visualization track encompassed by the CereVis workshop. A big thank you on behalf of all awardees and once again, sincere congratulations to all award winners!

CereVis Award Papers:

A Multiuser Virtual Reality Environment for Visualizing Neuroimaging Data, *by David Shattuck*

Augmenting Microsoft's HoloLens with Vuforia Tracking for Neuronavigation, *by Taylor Frantz; Bart Jansen; Johnny Duerinck; Jef Vandemeulebroucke*

Augmented reality guidance in cerebrovascular surgery using microscopic video enhancement, *by Reid Vassallo; Hidetoshi Kasuya; Benjamin Lo; Terry Peters; Yiming Xiao*

AE-CAI Award Papers:

Virtual Interaction and Visualisation of 3D Medical Imaging Data with VTK and Unity, *by Gavin Wheeler; Shujie Deng; Nicolas Toussaint; Kuberan Pushparajah; Julia Schnabel; John Simpson; Alberto Gomez Herrero*

ARssist: Augmented Reality on a Head-Mounted Display for the First Assistant in Robotic Surgery, *by Long Qian; Anton Deguet; Peter Kazanzides*

Endodontic Guided Treatment Using Augmented Reality on a Head-Mounted Display System, *by Ehsan Azimi; Tianyu Song; Chenglin Yang; Omid Dianat*

Fast and accurate vision-based stereo reconstruction and motion estimation for image-guided liver surgery, *by Andrew Speers; Burton Ma; William Jarnagin; Sharifa Himidan; Amber Simpson; Richard Wildes*

We hope that you will enjoy reading this Special Issue and we look forward to your continuing support and participation in future editions of the AE-CAI & CereVis workshops. Their continued success demands our ongoing commitment.

## Guest Editors:

Cristian A. Linte, Rochester Institute of Technology, USA

Marta Kersten-Oertel, Concordia University, Canada

Ziv Yaniv, National Institutes of Health and TAJ Technologies, USA

Yiming Xiao, Western University, Canada

## Guest Associate Editors:

Caroline Essert, University of Strasbourg, France

Pierre Jannin, University of Rennes I

Jonathan Lau, Western University, Canada

Philip Pratt, Imperial College London, UK

Ingerid Reinertsen, SINTEF, Norway

Hassan Rivaz, Concordia University, Canada

